# Practical Considerations for the Application of Nonlinear Indices Characterizing the Atrial Substrate in Atrial Fibrillation

**DOI:** 10.3390/e24091261

**Published:** 2022-09-08

**Authors:** Emanuela Finotti, Aurelio Quesada, Edward J. Ciaccio, Hasan Garan, Fernando Hornero, Raúl Alcaraz, José J. Rieta

**Affiliations:** 1 BioMIT.org, Electronic Engineering Department, Universitat Politecnica de Valencia, 46022 Valencia, Spain; 2 Arrhythmia Unit, Cardiology Department, General University Hospital Consortium of Valencia, 46014 Valencia, Spain; 3 Department of Medicine, Division of Cardiology, Columbia University Medical Center, New York, NY 10032, USA; 4 Cardiovascular Surgery Department, Hospital Clínico Universitario de Valencia, 46010 Valencia, Spain; 5 Research Group in Electronic, Biomedical and Telecommunication Engineering, University of Castilla-La Mancha, 16071 Cuenca, Spain

**Keywords:** atrial fibrillation, atrial arrhythmia, nonlinear indices, classification models, complex fractionated atrial electrogram, catheter ablation

## Abstract

Atrial fibrillation (AF) is the most common cardiac arrhythmia, and in response to increasing clinical demand, a variety of signals and indices have been utilized for its analysis, which include complex fractionated atrial electrograms (CFAEs). New methodologies have been developed to characterize the atrial substrate, along with straightforward classification models to discriminate between paroxysmal and persistent AF (ParAF vs. PerAF). Yet, most previous works have missed the mark for the assessment of CFAE signal quality, as well as for studying their stability over time and between different recording locations. As a consequence, an atrial substrate assessment may be unreliable or inaccurate. The objectives of this work are, on the one hand, to make use of a reduced set of nonlinear indices that have been applied to CFAEs recorded from ParAF and PerAF patients to assess intra-recording and intra-patient stability and, on the other hand, to generate a simple classification model to discriminate between them. The dominant frequency (DF), AF cycle length, sample entropy (SE), and determinism (DET) of the Recurrence Quantification Analysis are the analyzed indices, along with the coefficient of variation (CV) which is utilized to indicate the corresponding alterations. The analysis of the intra-recording stability revealed that discarding noisy or artifacted CFAE segments provoked a significant variation in the CV(%) in any segment length for the DET and SE, with deeper decreases for longer segments. The intra-patient stability provided large variations in the CV(%) for the DET and even larger for the SE at any segment length. To discern ParAF versus PerAF, correlation matrix filters and Random Forests were employed, respectively, to remove redundant information and to rank the variables by relevance, while coarse tree models were built, optimally combining high-ranked indices, and tested with leave-one-out cross-validation. The best classification performance combined the SE and DF, with an accuracy (Acc) of 88.3%, to discriminate ParAF versus PerAF, while the highest single Acc was provided by the DET, reaching 82.2%. This work has demonstrated that due to the high variability of CFAEs data averaging from one recording place or among different recording places, as is traditionally made, it may lead to an unfair oversimplification of the CFAE-based atrial substrate characterization. Furthermore, a careful selection of reduced sets of features input to simple classification models is helpful to accurately discern the CFAEs of ParAF versus PerAF.

## 1. Introduction

Atrial fibrillation (AF) is the most common cardiac arrhythmia diagnosed in clinical practice, with an estimated prevalence of about 1–2% of the general population and above 10% in the elderly [1]. Moreover, its prevalence is likely to double in the next 50 years as the population ages [2]. At least 15% of the budget in cardiac disease healthcare is earmarked to AF [3,4]. This disease is associated with an increased risk of stroke and congestive heart failure, so that AF patients have twice the risk of death as compared with healthy persons [5]. These facts make AF a major public health challenge, and its medical and economic aspects could worsen in the future [2]. From an electrophysiological viewpoint, AF is characterized by rapid and chaotic contractions of the atria, originating in disorganized atrial electrical activation [6]. As with many other arrhythmias, AF may require therapeutic intervention, even in patients who suffer no subjective discomfort [7]. Since Haissaguerre et al. reported the paramount relevance of the pulmonary veins (PV) in the initiation and maintenance of AF [8], the procedure of catheter ablation (CA) targeting PV foci, namely pulmonary vein isolation, has become such an effective therapy for AF [9], it is considered the first-line therapeutical alternative to pharmacological treatments.

Despite its high prevalence, the physiological mechanisms underlying AF are still pending to be completely understood, and the present therapeutic approaches to AF have major limitations [10]. During recent years, many efforts have addressed personalizing CA treatments by mapping the atrial electrophysiological substrate [11] and have been introduced with the aim to identify the arrhythmogenic atrial sites responsible for AF generation [12]. In this respect, atrial substrate characterization has been one of the most recent approaches aimed at reducing the limited clinical efficacy of the current therapeutic intervention, due to the major knowledge gaps in the mechanisms for sustaining AF [13].

In clinical practice, one of the goals for the characterization of the atrial substrate aims at discerning patients with paroxysmal AF (ParAF) versus persistent AF (PerAF) [14], as statistics have shown that the success rate of CA is dependent on the area of ablation and the AF type [15]. In fact, a high success rate is reached in ParAF just ablating the pulmonary veins (PVs), while for PerAF, the use of further ablations is required to achieve similar results [12]. This defines a challenge for the precise characterization of the atrial substrate aimed at optimally guiding the CA, where methodologies to distinguish between the complex fractionated atrial electrograms (CFAEs) of ParAF versus PerAF would be very interesting and useful for fast and efficient atrial substrate mapping methods [16]. CFAEs can be identified by the presence of multiple electrogram deflections without interruption, a baseline perturbation with continuous deflection [17], or a cycle length ≥120 ms that includes isoelectric intervals between deflections [18].

In the attempt to personalize AF treatment, nonlinear indices have been applied to CFAEs, which is aimed at quantifying atrial remodeling and the atrial electrophysiological substrate, supporting clinical management decisions, and suggesting the most appropriate approach for ablation procedures [19]. In this regard, different works have been published proposing several classification strategies based on nonlinear metrics as assessed via statistical tests, aimed at classifying ParAF versus PerAF via a CFAE analysis. In this respect, Ciaccio et al. measured the CFAE repetitiveness [16] and quantified the degree of morphological heterogeneity in CFAE deflections [20]; Acharya et al. adopted recurrence plots, the Recurrence Quantification Analysis, and entropy measures, proving that the underlying signal generation process of CFAEs in AF is somehow repetitive, even for sequences as short as one second [21]; Ndrepepa et al. and Ravi et al. used the AF cycle length to show that patients with persistent AF had shorter cycle lengths and a higher degree of disorganized activity than patients with paroxysmal AF [22,23]; and Sanders et al. employed a spectral analysis to identify localized sites of high-frequency activity, reporting different distributions in paroxysmal versus permanent AF [24].

However, the aforesaid prior studies do not respond to the clinical demand for easy and intuitive interpretation methods for CFAEs [25]. In fact, clinicians demand the use of straightforward classification models which are readily understandable, fed with features easily applicable to CFAEs. Furthermore, previous methods do not provide any signal quality or stability assessment applied to the variables and recordings used to develop the introduced models to discriminate between AF types, so that the robustness of the approaches previously introduced can be compromised. In fact, the prior reported results miss the assessment of the intra-recording and intra-patient stability of the analyzed data, as well as the CFAE signal quality. By omitting the study of the stability and the signal quality evaluation, two main issues may arise: first, averaging among recording places and AF types without having previously checked the intra-recording and intra-patient stability may lead to an oversimplification of the processes taking place at different regions of the atria; second, the inclusion of artifacted or noisy segments unlinked to the AF mechanism, such as drifts or very distorted recordings, due to bad contacts of the recording electrode on the atrial walls, may lead to biased and unreliable results in the characterization of the atrial electrophysiological substrate.

The present work has two principal objectives. On the one hand, to assess the stability of CFAEs with nonlinear indices, both intra-recording and intra-patient, and, on the other hand, to exploit nonlinear strategies and straightforward models to discriminate between the CFAEs of ParAF and PerAF from patients undergoing catheter ablation of the AF.

In the first part of the manuscript, the intra-recording and intra-patient stability of CFAEs have been assessed with the nonlinear indices of determinism (DET) of a Recurrence Quantification Analysis (RQA) and sample entropy (SE). Furthermore, the presence of artifacted or noisy segments in CFAEs has been considered as well, evaluating the consequences of their discarding in the final outcome. The idea behind it is that a discarding process to remove poor quality segments may benefit the intra-recording and intra-patient stability assessment. Moreover, this approach may decrease the differences in the DET and SE between the intra-patient recording places, thus helping to quantify the atrial substrate with reliable and representative values.

In the second part of the study, the exploitation of nonlinear strategies and the development of straightforward models to discriminate between ParAF and PerAF from the CFAEs of patients undergoing a CA of AF has been performed using, besides the nonlinear indices of DET and SE, the widely accepted indices of dominant frequency (DF) and the AF cycle length (AFCL). The indices extracted from CFAEs were processed and selected prior to being converted into features for coarse tree classification models. The assumption is that a thoughtful selection of reduced sets of indices, feeding straightforward classification models, would enable a more accurate discernment of ParAF versus PerAF CFAEs, thus providing a more understandable insight for atrial substrate evaluation and improved therapeutic decision for AF management.

## 2. Material and Methods

### 2.1. Database and Preprocessing

A total number of 212 electrograms of 16 s in length were acquired from ParAF and long-standing PerAF patients who were not under arrhythmic drug therapy, undergoing radiofrequency catheter ablation therapy at the cardiac electrophysiological laboratory of the Arrhythmia Unit in the General University Hospital Consortium of Valencia. The hospital’s Internal Review Board (IRB) approved acquisition and analysis of these retrospective data and informed consent was obtained from all individual participants included in the study. A total of 108 and 104 CFAEs were recorded from 27 patients for ParAF and 26 patients for PerAF, respectively, which were identified by observing the published criteria [16], from the four pulmonary veins (PV)—left superior (LSPV), left inferior (LIPV), right superior (RSPV), and right inferior (RIPV)—and the anterior (ANT) and posterior (POS) free wall of the left atrium.

If AF persisted for more than 10 min, then the electrograms were included for analysis and subjected to rectangular one-, two-, and four-second-length windowing, thus creating three distinct datasets. The first dataset consisted of 212 non-overlapping one-second-length sequences; the second dataset consisted of 212 non-overlapping two-second-length sequences, and the third dataset consisted of 212 non-overlapping four-second-length sequences. In the cases of ParAF with a baseline on sinus rhythm, AF was induced by rapid pacing at the coronary sinus or at the lateral wall of the right atrium, with a coupling interval range of 250–200 ms [16].

**Figure 1:**
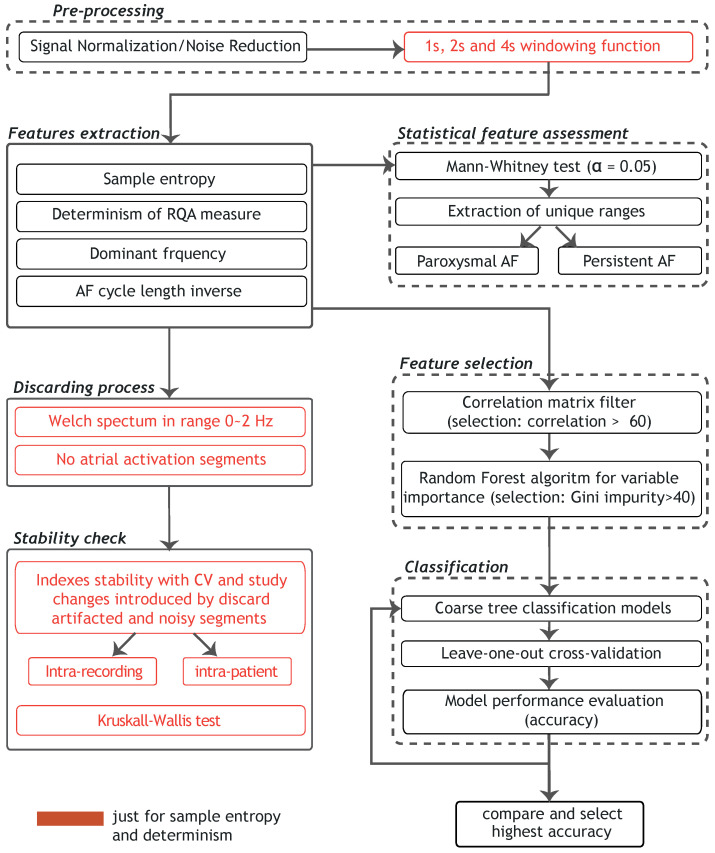
Flowchart describing the steps of operation of the proposed classification modeling.

The used catheter for signal acquisition was a 3.5 mm irrigated-tip radiofrequency ablation catheter and the procedure used 3D electroanatomic mapping (CARTO, Biosense-Webster Inc., Diamond Bar, CA). The protocol involved placing multipolar catheters in the right atrium and in the coronary sinus and a steerable circular catheter and “lasso” in the left atrium through transseptal puncture. All signals were band-pass filtered by the acquisition system (0.2–500 Hz) and digitized at a sampling frequency of 1 kHz by a Labsystem™ PRO EP recording system (Boston Scientific, Marlborough, MA, USA). The process applied to the analyzed signals is summarized in Figure 1. Finally, in order to remove the most relevant source of signal contamination from the cardiac electrophysiology laboratory, which is the ubiquitous powerline interference, a new method based on the stationary wavelet transform has been applied [26]. The use of this algorithm provided improved and truthful evaluation of CFAEs because it preserved signal morphology and did not add artificial fractionation to the electrograms [26].

### 2.2. CFAE Segment Discarding Process

CFAEs were visually inspected to evaluate their signal quality with two main purposes. On the one hand, it was to distinguish artifacts from real atrial activations, and, on the other hand, to identify very noisy CFAE segments that were useless for later stages. Signal segments of both these types were discarded via their corresponding quality information, as indicated in Figure 1.

The segment quality information of the *i*-th CFAE, with i=1,2,…,212, was stored in vector $q_{w}^{\left(i\right)}$, being *w* the segment length, which in this analysis can take the values of 1, 2, and 4, and having a dimension of $\frac{16}{w}$. Thus, each element of $q_{w}^{\left(i\right)}$ represented one segment of the length *w* of the *i*-th CFAE and could take one of two values: a value of 1 if the corresponding CFAE sequence had acceptable signal quality, and the value 0 if the segment had low quality. By building the vector in such a way, it was readily possible to turn off or discard low-quality segments by simply multiplying elements of $q_{w}^{\left(i\right)}$ for each *i*-th CFAE.

**Figure 2:**
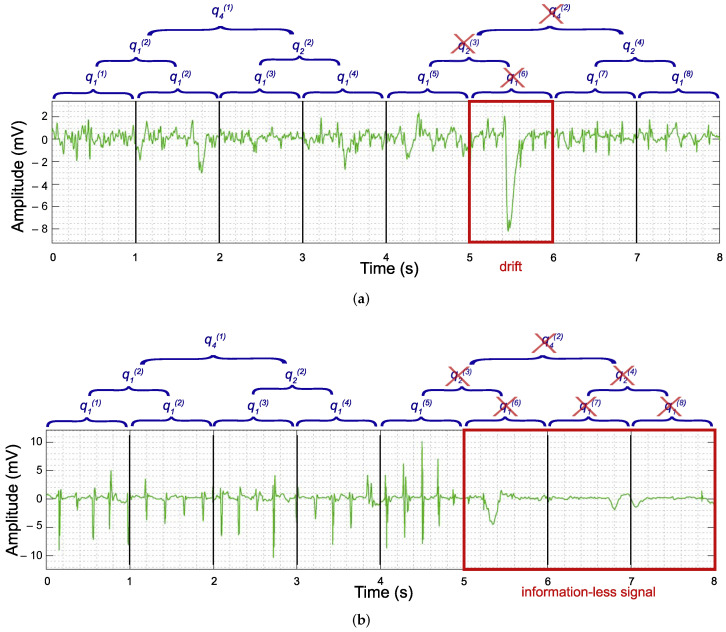
Examples of time-domain visual inspection to evaluate CFAE signal quality and the discarding process applied to 1, 2, and 4 s length segments. (**a**) First 8 seconds of a CFAE with an abnormal amplitude (drift) in the sixth second. The 1 s length element $q_{1}^{\left(6\right)}$ has been discarded. The goodness of the 2 s and 4 s length were derived and, respectively, elements $q_{2}^{\left(3\right)}$ and $q_{4}^{\left(2\right)}$ were also discarded. (**b**) First 8 seconds of a CFAE with signal lost, indicated by amplitude ranges near 0 mV. Elements $q_{1}^{\left(6\right)}$, $q_{1}^{\left(7\right)}$, and $q_{1}^{\left(8\right)}$ were discarded. The goodness of the 2 s length was affected and elements $q_{2}^{\left(3\right)}$ and $q_{2}^{\left(4\right)}$ were discarded. Finally, the 4 s length element $q_{4}^{\left(2\right)}$ was discarded as well.

Initially, the quality of 1 s length sequences was manually assessed, as they belong to the smallest window length (w=1), thus creating vector $q_{1}^{\left(i\right)}$. The process followed consisted of two steps: First, to detect low-frequency artifacts, the Welch spectrum of the 1 s length segments, using averaged modified periodograms [27], was computed and visualized, so that those segments presenting low-frequency peaks in the range of 0–2 Hz were observed in the time domain: the segments having an atrial activation with abnormal amplitude and/or shape were discarded, as shown in Figure 2a. Second, to recognize CFAE components lacking atrial activity, the amplitude ranges of each sequence were extracted and the segments presenting ranges near zero mV were discarded, as shown in Figure 2b. Clinicians participating in the study evaluated the quality of each segment with a double-check procedure, so that one of them marked the quality of a segment and other, randomly chosen, ratified the decision. Only segments with the two coincident opinions were considered in the study. The percentage of discards was assessed for each window length, recording place, and AF type.

Next, the goodness of the 2 s length sequences was derived from $q_{1}^{\left(i\right)}$, thus creating vector $q_{2}^{\left(i\right)}$. Consecutive elements of $q_{1}^{\left(i\right)}$ were regrouped to create mutually exclusive subsets of 2 elements. Then, the elements within each subset were multiplied and the outcome represented a new element of $q_{2}^{\left(i\right)}$. The subsets were created by following the time order of the segments, so that there was a time correspondence between datasets of different length. In such a way, for example, the first ($q_{1}^{\left(1\right)}$) and the second ($q_{1}^{\left(2\right)}$) elements of vector $q_{1}^{\left(i\right)}$ were multiplied to obtain the first element of $q_{2}^{\left(i\right)}$ ($q_{2}^{\left(1\right)}$), the third ($q_{1}^{\left(3\right)}$) and the fourth ($q_{1}^{\left(4\right)}$) elements of vector $q_{1}^{\left(i\right)}$ were multiplied to obtain the second element of $q_{2}^{\left(i\right)}$ ($q_{2}^{\left(2\right)}$), and so on. The same process was repeated also for the 4 s length datasets to obtain vector $q_{4}^{\left(i\right)}$.

Regrettably, with this method, some good but short sequences were discarded because just a part of the full 4 s length segment provoked its identification as a bad quality segment, causing a consequent reduction in available segments. The percentage of discarded segments was assessed for each window length dataset by counting the number of low-quality segments and dividing it by the total number of segments of the considered dataset. In addition, the embedded loss of information was quantified for the 2 and 4 s length datasets. Furthermore, the percentage of discarded segments and the consequent loss of information were assessed for the different recording places and types of AF analyzed.

### 2.3. Sample Entropy

The SE index assigned a non-negative value to the corresponding data series, reflecting the complexity of each sequence, with larger values corresponding to more irregularity in the data [28]. Mathematically, SE is defined as the negative natural logarithm of the probability that two sets similar for *m* data points are also similar for m+1 data points, given a tolerance distance *r*, with the exception of self-matches. Thus, given a time-series dataset of length N={x(n),n=1,…,N}, the k=1,…,N−m+1 vectors of length *m* are formed as $X_{m}\left(k\right)=\left\{x\left(k+i\right),i=0,\ldots ,m-1\right\}$. As a distance function $d[X_{m}\left(i\right),X_{m}\left(j\right)],\;\left(i\neq j\right)$ we used the Euclidean distance, computed as the maximum absolute distance between the scalar elements of the vectors. If the distance between the elements is below a tolerance *r*, then is counted as $B_{i}$. The counting element of different vectors is calculated and normalized as

(1) $$B^{m}\left(r\right)=\frac{1}{N-m}\sum_{i=1}^{N-m}\frac{B_{i}}{N-m-1}.$$

The repetition of the described process for vectors of length m+1 makes it possible to calculate $B_{m+1}\left(r\right)$; hence, SE can be defined as

(2) $$SE\left(m,r,N\right)=-\ln\left[\frac{B^{m+1(r)}}{B^{m}\left(r\right)}\right].$$

The maximum template length *m* was set to 2 samples and the tolerance *r* to 0.35 times the standard deviation of the segment. This setup was selected taking into consideration previous studies with an in-depth analysis of SE parameters, testing other combinations of *m* and *r* to achieve optimized classification of AF events, which are directly dependent on AF organization [29]. SE was applied to 1, 2, and 4 s length sequences datasets. The results are reported with range, mean, and standard deviation.

### 2.4. Recurrence Plots and Recurrence Quantification Analysis Measures

Recurrence plots (RPs) make possible the visualization of the recurrence behavior or states of the phase-space trajectory of natural processes, considered as dynamical systems [30]. Periodic signals, such as the electrocardiogram, are examples of recurrence [31], in which patterns are repeated over time. Mathematically, RPs can be defined as

(3) $$R\left(i,j\right)=\Theta (\epsilon -∥\vec{x}\left(i\right)-\vec{x}\left(j\right)∥),$$

where Θ is the function Θ: R→(0,1) and ϵ is a predefined distance.

In this work, RPs were used to visualize recurrent patterns within 1, 2, and 4 s length sequences. The phase-space reconstruction from a time series $u_{k}$ could be obtained following Takens’ embedding theorem [32] by using an embedding dimension *d* and a time delay τ. For the study, we selected the embedding dimension and the time delay using the False Nearest Neighbors (FNN) [33] and the mutual information (MI) [34] strategies, respectively. FNN and MI were applied to every segment of the subsets analyzed. In particular, after applying FNN and MI to every CFAE segment, we chose to reconstruct the phase space of all segments of a given length using the most frequent values as embedding dimension *d* and the time delay τ. Thus, given a CFAE, considered as a time series, its trajectory ${\left\{s_{i}\right\}}_{i=1}^{N}$, with *N* the number of considered states, was projected into a *d*-dimensional phase space in which the *i*-th point of the trajectory was represented as s(i). The vector of reconstructed state $\vec{s(t)}$ in the phase space at the time *t* is defined as

(4) $$\vec{s(t)}=\vec{s_{i}}=\left(u_{i},u_{i+\tau },\ldots ,u_{i+(d-1)\tau }\right),\;\;t=i\Delta t$$

Next, the RP was produced too. As shown in Equation (3), the RP was represented as an N×N array, where each element (i,j) is assigned a value of 1 if the distance between the point *i* and the point *j* of the trajectory $\vec{s(t)}$ is less than a specified threshold ϵ. For the study, we set the threshold ϵ as 10% of the mean of the phase-space diameter, and 0 otherwise, in accordance with previous works [35].

The Recurrence Quantification Analysis (RQA) measure selected for this study is the determinism [30], which quantifies the sequence predictability by measuring the percentage of recurrence points that belong to diagonal lines of a minimum length $l_{min}=50$.

### 2.5. Atrial Fibrillation Cycle Length

The AFCL is defined as the time gap between two consecutive atrial depolarizations, generally expressed in milliseconds [18]. The AFCL has been computed as the average distance between two consecutive atrial activations and serves to estimate the depolarization frequency of a certain region [36]. In this study, the AFCL was measured using a modified Botteron’s approach [37], decreasing its low cut-off frequency from 40 to 20 Hz, thus benefiting slow local activation detection. Moreover, high- and low-amplitude activations of CFAEs were equalized, thus facilitating the detection of low-amplitude activations [38]. The AFCL was computed on the 16 s length CFAEs at all the recording sites.

### 2.6. Dominant Frequency

The DF was measured by identifying the highest spectral peak in the electrophysiological frequency range of interest of 3–12 Hz from CFAE power spectra [39]. The power spectrum typically exhibits a distinct peak whose location determines the most common fibrillatory rate of nearby endocardial sites [40]. Most studies make use of Fourier-based spectral analysis, in which the signal is divided into shorter, overlapping segments, each segment subjected to windowing [27]. The global power spectrum is obtained by averaging the power spectra of the respective segments. In the present work, the DF was calculated using the Welch periodogram [27]. A Hamming window of 4096 points in length, a 50% overlapping between adjacent windowed sections, and an 8192-points Fast Fourier Transform (FFT) were used as computational parameters as suggested by previous works [41]. The largest amplitude frequency within the 3–12 Hz range was selected as the dominant frequency. The DF has been proven to be generally higher in PerAF, in accord with prior works [20]. Finally, the DF was computed on the 16 s length CFAEs at the different recording sites.

### 2.7. Intra-Recording and Intra-Patient Stability Assessment

The stability of the indices, both intra-recording and intra-patient, was checked for the 1, 2, and 4 s length datasets, using the coefficient of variation (CV) as a measure of the dispersion [42], as shown in Figure 1. The coefficient of variation, expressed as %, was calculated as CV=σ/μ, with σ the standard deviation and μ the mean of the index under study. The analyses were repeated, discarding artifacted or noisy CFAE segments, and any increase/decrease in the CV was assessed.

Furthermore, the Kruskal–Wallis test was selected to assess whether the index at the different recording places of a given patient originated from the same distribution [43]. Prior, the assumption of homoscedasticity was verified with the Breush–Pagan test [44]. The method was supported by comparing the median values of the groups both numerically and graphically, so that any inaccuracy could have been detected. The null hypothesis (H${}_{0}$) tested was that the mean ranks of DET (or SE) among the intra-patient recording places is the same. The acceptance of H${}_{0}$ proved that the atrial electrophysiological substrate condition is similar at different sites of the left atrium, as has been previously reported [21]. Contrarily, the rejection of H${}_{0}$ led to the conclusion that averaging among recording places provokes an oversimplification of the AF substrate taking place in the left atrium. The Kruskal–Wallis test was performed on the one-, two-, and four-seconds-length datasets, with and without discarded CFAE segments.

### 2.8. Statistical Feature Assessment

The Mann–Whitney test verified the null hypothesis that the indices were similar for ParAF and PerAF, with significance value set at 0.05 [45], as indicated in Figure 1. The test was performed for the indices at each recording site. For the three datasets, the *p*-values returned were averaged for recording places, and the resulted values are reported later in Section 3.

### 2.9. Feature Selection

In the present work, the features studied are the nonlinear indices of SE, DET, AFCL, and DF, computed in the PVs and in the anterior and posterior free wall of the left atrium. With the aim to remove data containing redundant or irrelevant variables without losing information, a feature selection procedure was applied to the data [46]. The adopted strategy combined two main techniques: the *filters* method, which selected the features independently from the predictor choice in a preprocessing step, and the *wrapper* method, which selected the features with machine learning techniques for classification, see Figure 1. For the *filters* method, we applied a filter to the correlation matrix, removing the most redundant variables with respect to the others, with a cut-off value of 0.60.

After correlation matrix filtering, the *wrapper* method selected was the Recursive Feature Elimination, which has been implemented via Random Forest (RF) for variable importance, providing a ranking of the most relevant features for classification [47]. The RF assigned to each variable a ranking from 0 to 100 based on the mean decrease Gini score, a measure that defines the contribution of each variable to the homogeneity of nodes and leaves in the RF. The higher the value of the Gini coefficient, the more important the feature and its value. As a trade-off empirical decision, the variables having scores higher than 40 were selected.

### 2.10. Classification of ParAF and PerAF with the Models

The classification of ParAF and PerAF was performed using coarse tree models together with leave-one-out cross-validation, as depicted in Figure 1. Each coarse tree model was built using one possible combination of the variables that passed the features selection process. In particular, for all segment lengths, all the possible subsets of features were used to determine the coarse tree providing the highest accuracy. Furthermore, aimed at further simplification of the classification process, a coarse tree model for each variable was created, having just one input feature. Regarding the cross-validation strategy, any recording of the patient under test was previously removed from the training set. Accuracy values obtained with the classification process were reported as percentages.

## 3. Results

**Figure 3:**
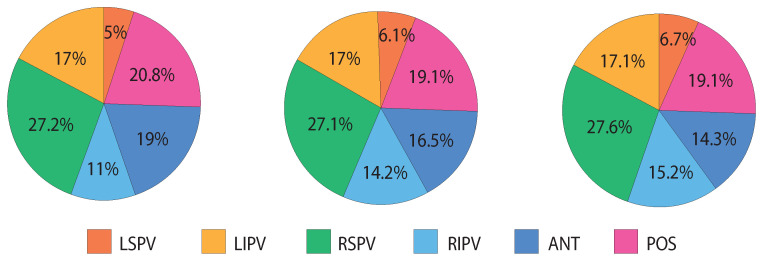
From left to right, discarding segments percentages distribution among recording places in 1 s length dataset, 2 s length dataset, and 4 s length dataset.

As a result of the application of the CFAE quality assessment, 5.2% of the one-second-length segments were discarded, while for the two- and four-second-length segments, the percentages were slightly higher, respectively, 8.6% and 12.5%, due to the aforesaid increased loss of discarded segments. Figure 3 shows the discarded segments distribution along the recording places in the datasets. In particular, the number of discards in the LSPV is quite low as compared with the other recording places and in contrast with the RSPV, in which the discards are more frequent. The proportion of the discarded segments in the paroxysmal and persistent AF patients is similar: for 1 s length segments, 46.4% of the discarded segments were in ParAF and 53.6% in PerAF; for the 2 s length segments, 44.4% were in ParAF and 55.6% in PerAF; and finally, for the 4 s length segments, 43.8% were in ParAF and 56.2% in PerAF.

Regarding the results of the stability, the averaged statistical descriptors (range, mean, and standard deviation) resulted by applying SE to the 1, 2, and 4 s length datasets, with and without discards, reported in Table 1. With the discards, the ranges were reduced as the lower boundary took greater values mainly due to the removal of the drifts, which generally presented high amplitude and low SE values. As a consequence, the standard deviations also diminished with the discards.

The statistical descriptors of the DET were computed in the same way as the SE, and the results are reported in Table 2. For the DET, the ranges were reduced with discards due to the upper boundary that took lower values, thus also decreasing the standard deviations. In fact, the DET and SE are complementary measures; one measures predictability, and the other, complexity.

The intra-recording analysis showed a significant variation in the CV(%) in any segment length, both for the SE and DET, as shown in Table 3. Discarding the segments benefited the stability, decreasing the CV, with deeper decreases for longer segments. These variations were on average of greater magnitude for PerAF (DET = 29.1%, SE = 37.6%) versus ParAF (DET = 19.6%, SE = 31.8%).

The intra-patient stability also provided large variations in the CV(%) for the DET and even bigger for the SE at any segment length, as shown in Table 4. In this case, discarding segments was useless and the CV provided similar variations.

The results of the Kruskal–Wallis test, as well as the visual inspection of the box plots computed for every recording place, every window length, and every index (72 plots not included here), suggests that the atrial electrophysiological substrate mostly differs at the recording places analyzed, and shows a great variability in the intra-patient indexing. For the 1 s length datasets, H${}_{0}$ was always rejected for the SE, while for the DET, it was accepted only once with no discard. For the 2 s length, H${}_{0}$ was still always rejected for the SE, while for the DET, it was accepted in two cases with no discard and in one case with discard. For the 4 s length, the null hypothesis was accepted in seven cases (one for the SE with discard, one for the DET with no discard, and five for the DET with discard); however, once the box plots were visualized to verify the accuracy of the results, the median values had demonstrated differences. The inaccuracy found for the 4 s length datasets are justified by the fact that the sample size is small, and therefore, the test does not follow a $\chi^{2}$ distribution.

With respect to the results for the discrimination between the CFAE of the ParAF versus PerAF, the mean and standard deviation values resulted by applying the SE and DET to the 1, 2, and 4 s length datasets, reported in Table 5 and organized by the AF type. The AFCL and the DF obtained from the 16 s length CFAEs demonstrated values of AFCL with an average and standard deviation of 7.75 ± 1.56 in ParAF and 7.01 ± 1.46 in PerAF, while the DF had, respectively, an average and a standard deviation of 5.59 ± 1.36 in ParAF and 6.20 ± 1.08 in PerAF.

As shown in Table 6 for the SE and DET, the Mann–Whitney test rejected the null hypothesis in most of the recording places, with particularly low *p*-values in the right superior pulmonary vein (RSPV), while in the right inferior pulmonary vein (RIPV), the null hypothesis was accepted, which suggests the presence of a similar atrial electrophysiological substrate in both AF types. For the AFCL, the *p*-values were always higher than the significance level of 0.05, except for the RSPV. Finally, the statistical tests run for the DF showed no differences between ParAF versus PerAF except in the left superior pulmonary vein (LSPV) and in the posterior free wall of the left atrium (POS).

**Figure 4:**
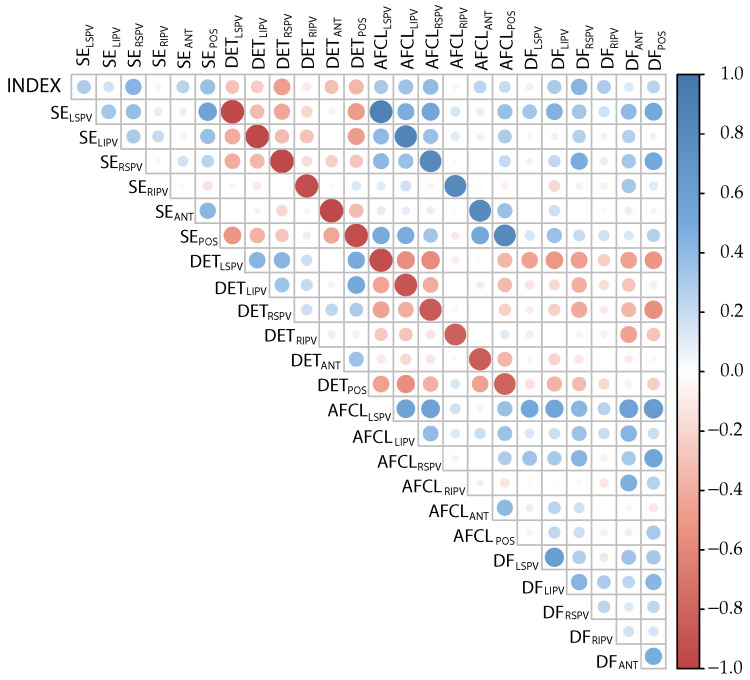
Correlation matrix showing the degree of bound between indices, applied to the considered recording places. The variable TYPE was assigned a value 0 if the patient is paroxysmal, and 1 if the patient is persistent AF.

The correlation matrix obtained by averaging the correlation values of the 1, 2, and 4 s length datasets is shown in Figure 4. In particular, a strong negative correlation between the index pairs of the SE-DET and AFCL-DET recorded at the same recording places was observed, while a strong positive correlation appeared between the paired SE-AFCL measured at the same recording place.

The application of the correlation matrix filter to 1 and 2 s length datasets removed the indices DF${}_{\mathrm{LIPV}}$ and entirely removed the indices of the DET and AFCL, due to their strong correlation with the SE. For the 4 s length dataset, the subset of indices kept was SE${}_{\mathrm{LSPV}}$, SE${}_{\mathrm{LIPV}}$, SE${}_{\mathrm{RSPV}}$, SE${}_{\mathrm{RIPV}}$, SE${}_{\mathrm{POS}}$, AFLC${}_{\mathrm{ANT}}$, DF${}_{\mathrm{LSPV}}$, DF${}_{\mathrm{RSPV}}$, DF${}_{\mathrm{RIPV}}$, DF${}_{\mathrm{ANT}}$, and DF${}_{\mathrm{POS}}$.

**Figure 5:**
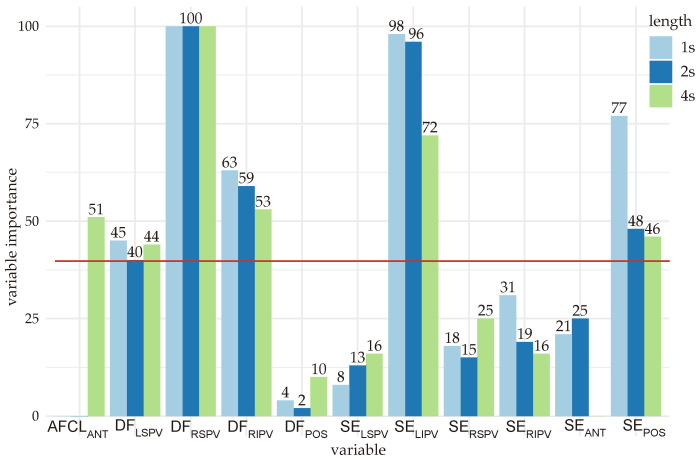
Variable importance ranked with the Random Forest algorithm for 1, 2, and 4 s length analyses. The cut-off value of 40 is shown in red.

For the three datasets, the variables scoring provided by the Random Forest is presented in Figure 5. For the 1 and 2 s length datasets, the variables ranked with a score higher than 40 were DF${}_{\mathrm{LSPV}}$, DF${}_{\mathrm{RSPV}}$, DF${}_{\mathrm{RIPV}}$, SE${}_{\mathrm{LIPV}}$, and SE${}_{\mathrm{pos}}$, while in the 4 s length dataset, the AFLC${}_{\mathrm{ANT}}$ was also included as it surpassed the threshold value.

After testing all the possible combinations of the high-ranked features, the group SE${}_{\mathrm{POS}}$, DF${}_{\mathrm{LSPV}}$, DF${}_{\mathrm{RSPV}}$, and DF${}_{\mathrm{RIPV}}$ provided the best classification performance to discriminate between the CFAE of ParAR and PerAF with an accuracy of 88.3% for all the segment lengths. The same accuracy was reached by also adding SE${}_{\mathrm{LISP}}$ to the group. However, in order to simplify the model as much as possible, it was decided to consider the minimal set of features as optimal. The accuracy values of the models built with the other possible combinations of the highest-ranked features (score > 40) had a mean and standard deviation of 70.7 ± 8.7% for the 1 s length, 70.8 ± 8.3% for the 2 s length, and 69.2 ± 8.8% for the 4 s length, which is a significant reduction in the accuracy. Finally, the highest accuracy achieved by a single index was provided by DET${}_{\mathrm{LIPV}}$ with 82.2% for any segment length as well, while the averaged single accuracies reached by the other indices were 60.2 ± 11% for the 1 s length, 54.7 ± 13.7% for the 2 s length, and 57.3 ± 11.4% for the 4 s length.

## 4. Discussion and Conclusions

The present work demonstrates that the intra-recording and intra-patient stability assessment of CFAEs is significantly benefited by the exclusion of artifacted or noisy segments, thus helping to quantify the atrial electrophysiological substrate with reliable and representative values. By contrast, previous studies may lead to an oversimplification of the processes taking place at different regions of the atria, thus providing biased and unreliable results in the characterization of the atrial substrate.

To this respect, the stability of the CFAE and their corresponding nonlinear indices are significantly influenced by the length of the analyzed segment, and specifically by the recording site within the left atrium (see Figure 3). In this regard, the number of discards in the LSPV have been quite low compared to the other recording places. In contrast, the RSPV has been the recording site in which the discards have been more frequent. This fact could be due to the difficulties in reaching the RSPV using a Lasso^TM^ catheter (Biosense-Webster, Diamond Bar, CA, USA), such as in the present study, in comparison with basket catheterization. In fact, catheters such as the Constellation^TM^ (Boston Scientific, Natick, MA, USA) have the advantage to better fit in most veins, adapting to size and anatomical form [48], their main disadvantage being the higher cost [49,50].

The introduction of discards provoked the reduction in ranges and standard deviations both in the SE and DET (Table 1 and Table 2), thus showing that mapping CFAE with contact catheters on a beating heart is a delicate task [48]. Furthermore, the discarding process benefited more the intra-recording stability (Table 3) than the intra-patient stability (Table 4), so that the CV varied more significantly in the first case. Nonetheless, the continued high variability of the CV suggests that averaging data in the same recording (intra-recording), as well as among recording places (intra-patient), may lead to an unfair oversimplification of the CFAE-based atrial electrophysiological substrate characterization, which has not been considered in many previous studies. In particular, in the intra-patient analyses, the visualized box plots exhibited many instances in which just a part of the recording places presented similar atrial electrophysiological properties as identified by like DET and SE values, enhancing the conclusion that averaging causes a loss of singularity of the electrophysiological substrate at the different atrial sites, which is the basis for the development of personalized catheter ablation procedures for AF treatment.

Unlike other complicated previously published models, this work has also proved that it is possible to develop straightforward solutions for clinical practice able to discriminate between ParAF and PerAF from the CFAEs of patients undergoing catheter ablation, thus providing a more understandable insight for atrial substrate evaluation and improved therapeutic decisions for AF management. To this respect, the SE mean values diminished with the segment length (Table 5), with greater values in PerAF as compared to ParAF, thus reflecting a higher degree of disorganization in PerAF, which is in agreement with a previous study [22]. Similarly, the DET values increased with the segment length, showing greater values in ParAF versus PerAF, thus highlighting ParAF as more predictable than PerAF.

The computation of the AFCL from the 16 s length CFAEs resulted in higher values for ParAF than for PerAR, as well as in lower values of DF for ParAF than for PerAF, as has been reported in prior work [40]. However, the discussion remains open in this respect, because other studies have reported DF peak frequencies higher in ParAF than in PerAF [51].

The study of the correlation between the indices and recording sites is illustrative (see Figure 4) because it showed a high correlation between the SE and DET in the same recording site. Similarly, a high correlation was also observed between the AFCL and SE, and the AFCL and DET, thus indicating that these three indices maintain a strong relationship between each other, linking the linear and the nonlinear domain. Finally, as expected, the AFCL and DF were also highly correlated. Altogether, these high correlations indicate that the selection of such indices for a substrate assessment is the right choice because they have been able to capture the essence of the atrial electrophysiological substrate.

The study of the discriminatory power of DF${}_{\mathrm{RIPV}}$ using statistical tests led to the wrong conclusion that the index is not discriminative, as the null hypothesis of no differences between ParAF and PerAF was accepted. Contrarily, the feature selection ranked the DF${}_{\mathrm{RIPV}}$, together with SE${}_{\mathrm{POS}}$, DF${}_{\mathrm{LSPV}}$, and DF${}_{\mathrm{RSPV}}$, as the set of most important variables to discriminate between the CFAEs of the ParAF and PerAF, reaching the highest accuracy of 88.3%. Therefore, the careful selection of limited sets of indices feeding straightforward classifiers are able to discriminate accurately between the CFAE of different AF types. Furthermore, the use of just one nonlinear index, such as DET${}_{\mathrm{LIPV}}$, provided a classification accuracy as high as 82.2% for any segment length. This result can serve as a starting point to prove that simple classification models, which are readily understandable, can be built to provide improved methodologies for atrial substrate characterization in AF.

The proposed analysis has also some limitations that merit consideration. Firstly, the study has been carried out using a limited set of data which, lately, has been reduced more due to the discarding process. Obviously, in order to obtain more generalizable results, a wider database with many more patients would be desirable. In this regard, our group is now working toward the expansion of the database for future studies. Secondly, regarding the creation of the classification models, the generalization of the results for the classification between the ParAF and PerAF could provoke model overfitting, especially using the Random Forest algorithm. However, given that the accuracy obtained with a single index has been high enough (82.4%) with respect to the accuracy provided by the most performing model (88.2%), it is reasonable to consider that overfitting has a reduced effect in overall classification performance.

As an overall conclusion, the observed high variability of the CFAE has shown that averaging data in one recording place or among different recording places may lead to an unfair oversimplification of the CFAE-based atrial electrophysiological substrate characterization. Furthermore, a thoughtful selection of the limited combinations of features feeding straightforward classification models are able to discriminate accurately between the CFAEs of the ParAF and PerAF, thus providing improved therapeutic decision making for AF management, as well as clearer insight concerning the evaluation of the atrial substrate.

## Figures and Tables

**Table 1:** Sample entropy statistical descriptors analysis results for 1, 2, and 4 s length datasets, with and without discards. The values reported were obtained by averaging among CFAE.

Length	No Discard		Discard	
	Range	Mean ± Std	Range	Mean ± Std
**h**	[0.004–0.362]	0.137 ± 0.033	[0.013–0.362]	0.138 ± 0.032
**2 s**	[0.007–0.312]	0.134 ± 0.025	[0.018–0.312]	0.135 ± 0.023
**4 s**	[0.008–0.300]	0.132 ± 0.017	[0.020–0.300]	0.134 ± 0.014

**Table 2:** Determinism statistical descriptors analysis results for 1, 2, and 4 s length segments, with and without discards. The values reported were obtained by averaging among CFAE.

Length	No Discard		Discard	
	Range	Mean ± Std	Range	Mean ± Std
**1 s**	[0.075–0.998]	0.561 ± 0.120	[0.075–0.963]	0.559 ± 0.113
**12**	[0.122–1.000]	0.600 ± 0.101	[0.122–0.958]	0.595 ± 0.091
**4 s**	[0.092–1.000]	0.631 ± 0.072	[0.124–0.942]	0.621 ± 0.058

**Table 3:** Intra-recording CV of DET and SE for 1, 2, and 4 s length segments, and the respective variation of the CV (ΔCV) introduced by discarding low-quality segments.

	1 s	ΔCV 1 s	2 s	ΔCV 2 s	4 s	ΔCV 4 s
**DET**	23.3%	−15.6%	19.1%	−22.8%	13.3%	−47.9%
**SE**	26.6%	−16.1%	20.5%	−20.1%	13.9%	−42.2%

**Table 4:** Intra-patient CV of DET and SE for 1, 2, and 4 s length segments and the respective variation of the CV (ΔCV) introduced by discarding low-quality segments.

	1 s	ΔCV 1 s	2 s	ΔCV 2 s	4 s	ΔCV 4 s
**DET**	23.9%	+2.0%	24.8%	+3.5%	24.1%	+7.7%
**SE**	34.2%	+0.5%	34.8%	−0.1%	35.9%	−0.3%

**Table 5:** Sample entropy and determinism mean and standard deviation values for datasets of 1, 2, and 4 s length segments. The values reported were obtained by averaging among CFAE.

Length	SE		DET	
	ParAF	PerAF	ParAF	PerAF
**1 s**	0.123 ± 0.042	0.148 ± 0.057	0.608 ± 0.135	0.524 ± 0.160
**2 s**	0.120 ± 0.043	0.145 ± 0.056	0.656 ± 0.140	0.556 ± 0.177
**4 s**	0.117 ± 0.043	0.143 ± 0.057	0.688 ± 0.135	0.585 ± 0.179

**Table 6:** Mann–Whitney test *p*-values obtained, for each recording place, by averaging the *p*-values returned in the test performed for the three segment-length datasets.

Recording Place	SE	DET	AFCL	DF
LSPV	p=0.0006	p=0.0001	p>0.0500	p>0.0500
LIPV	p=0.0253	p=0.0080	p>0.0500	p=0.0287
RSPV	p<0.0001	p<0.0001	p=0.0192	p=0.0007
RIPV	p>0.0500	p>0.0500	p>0.0500	p=0.0035
ANT	p=0.0035	p=0.0016	p>0.0500	p=0.0387
POS	p=0.0002	p=0.0006	p>0.0500	p>0.0500

## Data Availability

The data supporting reported results and presented in this study are available on request from the corresponding author.
